# Supplementary material for: Analysis of PPARγ Signaling Activity in Psoriasis
Source: Int J Mol Sci. 2021 Aug 10;22(16):8603. doi: 10.3390/ijms22168603 (PMC8395241; doi:10.3390/ijms22168603)
Supplement: Supplementary file 1 [file ijms-22-08603-s001.zip › Supplemental materials_Analysis of PPARg signaling activity in psoriasis/Pathway models/Models images and html files/Model of PPARG signaling in psoriasis/57337.html]

diacylglycerol


# Small Molecule diacylglycerol

|  |  |
| --- | --- |
| URN | urn:agi-smol:diacylglycerols |
| Total Entities | 7 |
| Connectivity | 2423 |
| Name | diacylglycerol |
| Description | diacylglycerol |
| Class | Endogenous compound |

---

|  |  |
| --- | --- |
| ChildConcepts | 1,2-dioctanoylglycerol |
|  | dioleoylglycerol |
|  | 1-oleoyl-2-acetylglycerol |
|  | monogalactosyl diacylglycerol |
|  | diacylglycerol derivatives |
|  | dicaprin |
|  | dioctanoylglycerol |

---

|  |  |
| --- | --- |
| Pathway | Skin Aging |
|  | mTOR Signaling Activation by Fatty Acids and Glucose |
|  | Ghrelin and Nesfatin-1 Regulate Stomach Functional State and Satiety |
|  | Leptin and CCK8 Activate Nodose Ganglia Neuron |
|  | Ghrelin and SST Regulate GH1 Release in Hypothalamus |
|  | P-cell Activation |
|  | D-cell: Somatostatin Release |
|  | A-cell (X-like): Ghrelin and Nesfatin-1 Synthesis |
|  | G-cell: Gastrin Secretion |
|  | ECL-cell: CHGA and Histamine Synthesis |
|  | Insulin Influence on Lipogenesis |
|  | Dopamine Receptors Signaling |
|  | Serotonin Receptors Signaling |
|  | Catecholamines Secretion from Adrenal Gland |
|  | Thyroid Hormones Common non-Genomic Effects in Hyperthyroidism |
|  | Norepinephrine Release Regulation |
|  | Dopamine Synthesis and Release |
|  | CRH Secretion Regulation |
|  | Prolactin Production |
|  | Oxytocin Action in Mammary Gland |
|  | Thrombopoietin Receptors Signaling in Platelet Maturation |
|  | Mineralcorticoids Secretion Regulation |
|  | T-Cell Dependent B-Cell Activation |
|  | T-Cell Independent B-Cell Activation |
|  | V(D)J Recombination Activation |
|  | Natural Killer Cell Activation through C-Type Lectin-like Receptors |
|  | Natural Killer Cell Activation through ITAM-Containing Receptors |
|  | Natural Killer Cell Activation through ITSM-Containing Receptors |
|  | Natural Killer Cell Activation |
|  | Peripheral T-Cell Tolerance |
|  | CD8+ T-Cell Activation |
|  | Th1-Cell Differentiation |
|  | Treg-Cell Differentiation |
|  | Th2-Cell Differentiation |
|  | T-Cell Receptor Signaling |
|  | Th17-Cell Differentiation |
|  | CXC Chemokine Receptor Signaling |
|  | CC Chemokine Receptor Signaling |
|  | Plasmin Effects in Inflammation |
|  | Platelet Activation via Adhesion Molecules |
|  | Scavenger Receptors in Platelet Activation |
|  | Scavenger Receptors in Platelet Aggregation |
|  | Proplatelet Maturation |
|  | Bradykinin Effects in Inflammation |
|  | Platelet Activation via GPCR Signaling |
|  | Toll-like Receptor Independent Sterile Inflammation |
|  | Lipoxin A4/FPR2-Related Neutrophil Depression |
|  | Annexins in Inflammation Resolution |
|  | Eat me Signal: Apoptotic Cell Induces Phagocytosis |
|  | Leukotriene Effect on Vascular Endothelial Cell Response |
|  | Eicosanoids in Myeloid Cell Activation |
|  | Leukotriene Effect on Airway Smooth Muscle Response |
|  | Neutrophil Activation via LTB4/Polyisoprenyl Phosphate Signaling |
|  | Basophil Activation |
|  | Eosinophil Survival by Cytokine Signaling |
|  | Eosinophil Activation |
|  | Mast-Cell Activation via IgE Signaling |
|  | Mast-Cells De Novo Synthesized Mediators via IgE Independent Signaling |
|  | CD16/CD14 Proinflammatory Monocyte Activation |
|  | Macrophage M2-Related Phagocytosis |
|  | Neutrophil Recruitment and Priming |
|  | Neutrophil Activation via FCGR3B |
|  | Neutrophil Degranulation via FPR1 Signaling |
|  | CR3 Mediated Phagocytosis in Neutrophils and Macrophages |
|  | Neutrophil Chemotaxis |
|  | Neutrophil Activation via Adherence on Endothelial Cells |
|  | SNS Related Adrenal Cortex Response |
|  | Vascular Endothelial Cell Activation by NO |
|  | Vascular Smooth Muscle Cell/Pericyte Migration and Proliferation |
|  | Vascular Endothelial Cell Activation by Cytokines |
|  | Vascular Endothelial Cell Activation by Blood Coagulation Factors |
|  | Vascular Endothelial Cell Activation by Growth Factors |
|  | Vascular Endothelial Cell Permeability Activation |
|  | Lipolysis Regulation in Adipocyte |
|  | Lipogenesis Regulation in Adipocyte |
|  | Potassium Flux Regulation |
|  | GFs/TNF -> Ion Channels |
|  | Nociception Expression Targets Signaling |
|  | Vascular Motility |
|  | Ca2+ Flux Regulation |
|  | TAAR1 -> Neurotransmitter Uptake |
|  | NPY1R -> CRH/POMC Production |
|  | NTSR1 Expression Targets |
|  | EDNRA/B -> Vascular Motility |
|  | CCKBR -> Neurotransmitter Uptake |
|  | ADORA3 -> Mast-Cell Degranulation |
|  | ADRA1 -> Vasoconstriction |
|  | ADRA1A -> IL6 Production |
|  | ADRA1 -> Ion Channels |
|  | ADRA2C/ADRB2 -> Vasoconstriction |
|  | ADRA2C/ADRB2 -> Synaptic Endocytosis |
|  | ADRA1 -> Prostaglandin Generation |
|  | BDKRB1/2 -> Ion Channels |
|  | BDKRB1/2 -> Prostaglandin Generation |
|  | CNR1/2 -> Membrane Transport |
|  | CNR1/2 -> IL1B/2/4/6/10 Production |
|  | DRD3 -> Dopamine Uptake |
|  | DRD2 Expression Targets |
|  | DRD2/4 -> Membrane Transport |
|  | GALR1/2/3 -> POMC/NPY Production |
|  | GALR1/2/3 -> Neurotransmitter Metabolism |
|  | CHRM1 -> IL2 Production |
|  | CHRM1/2/3/5 -> Ion Channels |
|  | CHRM1/2/3 -> Vascular Motility |
|  | OPRK -> Pain Perception |
|  | PTGFR -> Vasoconstriction |
|  | PTGIR -> IL6 Production |
|  | PTGER1/4 -> Vascular Motility |
|  | PTGER2/3 -> Vascular Motility |
|  | PTGER2/3 -> Inflammation-Related Expression Targets |
|  | HTR1 -> IL6 Production |
|  | HTR5 -> TNF Production |
|  | HTR2 -> Membrane Transport |
|  | HTR1 -> Membrane Transport |
|  | TACR1 -> Membrane Transport |
|  | GRM1/5 (Postsynaptic) -> Ion Channels |
|  | P2RY11/13/14 -> IL8/10 Production |
|  | NTRK1/2/3 -> Acetylcholine Production |
|  | Luteinizing Hormone in Oocyte Meiotic Resumption |
|  | Endocannabinoids Role in Sleep Regulation |
|  | Circadian Clock Genes in Suprachiasmatic Nuclei Neurons |
|  | Hypocretin (Orexin) in Arousal Regulation |
|  | Retinal Ganglion and Suprachiasmatic Nucleus in Melatonin Synthesis Regulation |
|  | RHO Signaling (Light Signal Termination and Photoreceptor Recovery) |
|  | Vitamine D Deficite and Dentin Formation |
|  | G0/G1 Phase Transition |
|  | Gap Junction Assembly |
|  | beta-Cell Granules Exocytois |
|  | Th1-Cells Activation and Proliferation in Atherosclerosis |
|  | Dilated Cardiomyopathy |
|  | Cardiomyocyte Hypertrophy |
|  | Hypertrophic Cardiomyopathy |
|  | Endothelin Signaling in Arterial Hypertension |
|  | Aldosterone Synthesis Acute Regulation |
|  | Endothelial Cell Dysfunction in Arterial Hypertension |
|  | Aldosterone Synthesis Chronic Regulation |
|  | Smooth Muscle Cell Dysfunction in Arterial Hypertension |
|  | TRPM4/6/7/8 Signaling in Arterial Hypertension (Hypothesis) |
|  | Smooth Muscle Cell Dysfunction in Pulmonary Hypertension |
|  | Myocardial Remodeling |
|  | Lymphocyte Mediated Myocardial Injury in Myocarditis |
|  | Vascular Reactivity in Raynaud Disease |
|  | Cold Stress and Adrenoceptor alpha 2C Signaling in Raynaud Disease |
|  | NO Synthesis in Early Vasospasm |
|  | Neurons Necrosis Caused by Energy Deficiency |
|  | Contraction Due Vasospasm |
|  | Sebocyte Proliferation in Acne Vulgaris |
|  | Androgens in Sebocyte Maturation |
|  | Atopic Dermatitis Onset |
|  | Mast-Cells Activation in Atopic Dermatitis |
|  | Cell Cycle Dysregulation in Melanoma |
|  | WNT Signaling in Melanoma |
|  | Melanoma |
|  | Apoptosis Block in Melanoma Cell |
|  | MITF as a Regulator of Melanoma Cell Development |
|  | Apoptotic Keratinocytes Clearance Recession in Systemic Lupus Erythematosus |
|  | Th0-Cell Aberrant Activation in Systemic Lupus Erythematosus |
|  | Th17-Cell Function in Systemic Lupus Erythematosus |
|  | B-Cell Activation in Systemic Lupus Erythematosus |
|  | Th2-Cell Function in Systemic Lupus Erythematosus |
|  | Oxidative Stress in Vitiligo |
|  | Acute Cytotoxic CD8+T-Cell Response against Melanocytes |
|  | Vitiligo |
|  | T-Cell Cytotoxic Response against Melanocytes in Vitiligo |
|  | Apoptosis of Melanocyte in Vitiligo |
|  | Humoral Immunity in Vitiligo |
|  | Treg-Cell Function in Diabetes Mellitus Type 1 |
|  | Natural Killer T-Cell Roles in Diabetes Mellitus Type 1 |
|  | non-Suppressive Treg-Cell in Diabetes Mellitus Type 1 |
|  | TLR2 Signaling in Treg-Cell in Type 1 Diabetes (Animal Model) |
|  | Natural Killer Cell in Diabetes Mellitus Type 1 |
|  | Peripheral Tolerance to Autoantigens Recession in Diabetes Mellitus Type 1 |
|  | Treg-Cell Activation in Diabetes Mellitus |
|  | MGAM Inhibitors |
|  | Protein Kinase C in Diabetic Microangiopathy |
|  | Peripheral Tissue Microangiopathy |
|  | Tubular Cell Dysfunction in Progressive Diabetic Nephropathy |
|  | Podocyte Dysfunction in Diabetic Nephropathy |
|  | Endothelial Cell Dysfunction in Early Diabetic Nephropathy |
|  | Mesangial Cell Dysfunction in Diabetic Nephropathy |
|  | Hyperosmotic Stress in Diabetic Neuropathy |
|  | Oxidative Stress in Diabetic Neuropathy |
|  | Microangiopathy in Diabetic Neuropathy |
|  | Proliferative Diabetic Retinopathy |
|  | Adipokines and Cytokines in Insulin Resistance in Skeletal Muscles |
|  | Insulin Resistance in Myocytes Induced by Obesity |
|  | Adipose Tissue Inflammation Provokes Insulin Resistance |
|  | Insulin Resistance in Hepatocytes |
|  | PNPLA3 in non-Alcoholic Fatty Liver Disease |
|  | G-Proteins Signaling in Insulin Secretion in beta-Cell |
|  | Olanzapine Effect on Insulin Secretion in Diabetes Mellitus |
|  | Hyperparathyroidism, Neonatal Severe |
|  | RET1 Mutation in Hyperparathyroidism |
|  | Bone Remodeling in Hyperthyroidism |
|  | Cardiovascular Effects in Hyperthyroidism |
|  | Thyroid Dysfunction |
|  | GNRH Release Impairment in Hypogonadotrophic Hypogonadism |
|  | GNRH Signaling Impairment in Hypogonadotrophic Hypogonadism |
|  | KISS1 Production Impairment in Hypogonadotropic hypogonadism |
|  | Hypoparathyroidism, Primary |
|  | Hypothyroidism, Secondary (Central) |
|  | Thyroid Stimulating Hormone (TSH) Resistance in Congenital Hypothyroidism |
|  | Overt Hypothyroidism, Primary |
|  | Thyroid Stimulating Hormone (TSH) Secretion in Overt Hypothyroidism |
|  | Central Hypothyroidism |
|  | Tertiary Hypothyroidism |
|  | Fatty Acids in Hypertension and Atherosclerosis Development |
|  | Adipocyte Hypertrophy and Hyperplasia |
|  | Prolactinoma Overiew |
|  | CD4+T-Cell Response in Celiac Disease |
|  | CD8+ T-Cell Response in Celiac Disease |
|  | Metastatic Colorectal Cancer |
|  | OipA Signaling in Helicobacter Pylori Infection |
|  | CagA Phosphorylation Dependent Signaling |
|  | EDN, NRG, NRTN, and GDNF/RET Signaling in Hirschsprung Disease |
|  | Th2-Cell Function in Ulcerative Colitis |
|  | T-Cell Cytotoxic Response in Ulcerative Colitis |
|  | Antiphospholipid Antibodies in Platelets and Coagulation Cascade |
|  | B-Cell Chronic Lymphocytic Leukemia |
|  | Diffuse Large-B-Cell Lymphoma |
|  | Diffuse Large-B-Cell Lymphoma ABC Subtype |
|  | Hodgkin Lymphoma |
|  | Mantle Cell Lymphoma |
|  | mTOR/NF-kB/BCR Signaling Disregulation in Mantle Cell Lymphoma |
|  | Mucosa-Associated Lymphoid Tissue (MALT) Lymphoma |
|  | Splenic Marginal Zone Lymphoma |
|  | Osteoclast Activation in Multiple Myeloma |
|  | Platelet and Coagulation Cascade Activation by Antiphospholipid Antibodies |
|  | T-Cell Acute Lymphoblastic Leukemia |
|  | TRPM7/8 in Pancreatic Adenocarcinoma |
|  | Eosinophil Survival in Asthma |
|  | Eosinophil Activation and Degranulation in Asthma |
|  | Airway Smooth Muscle Cell High Rate Proliferation |
|  | IgE Induces Airway Smooth Muscle Cell Proliferation |
|  | Airway Smooth Muscle Cell Contraction |
|  | Goblet-Cell Exocytosis in Asthma |
|  | Neutrophil Degranulation via FPR1/IL8 |
|  | Basophil Activation in Asthma |
|  | Mast-Cell Activation in Asthma |
|  | Mast-Cell Related Chemokines Signaling in Asthma |
|  | Antigen-Presenting Cell Role in Asthma |
|  | Th17-Cell Differentiation in Asthma |
|  | Th2-Cell Response in Asthma |
|  | CFTR Facilitates Oxidative Stress in Airway Epithelium in CF |
|  | Airway Epithelial Cell Dysfunction |
|  | CFTR Expression in Epithelial Cells (Class I Mutations) |
|  | CFTR Related Ion-Channel Dysfunction in Cystic Fibrosis Airway Epithelium (Class III Mutations) |
|  | Steroidogenesis Impairement in Polycystic Ovary Syndrome |
|  | Ovulation Block |
|  | FSH Action in Polycystic Ovary Syndrome |
|  | Th17-Cell and Th1 Immune Responsein Psoriatic Arthritis |
|  | PTPN22 Role in Rheumatoid Arthritis |
|  | B-Cells Function in Systemic Scleroderma |
|  | Th2-Cells Function in Systemic Scleroderma |
|  | Skin Fibrosis |
|  | Dentin Formation Disruption |
|  | CCR5 Signaling in Macrophages |
|  | CD4+ T-Cell Function Decline in HIV |
|  | CD4+ T-Cell Death in HIV |
|  | Majeed Syndrome |
|  | Ca2+ Overload in Muscular Dystrophies |
|  | Dystrophin Glycoprotein Complex Signaling in Muscular Dystrophies |
|  | BDNF Upregulation Triggered by Seizures |
|  | Apoptosis and Immediate Early Gene Activation in Epileptiform Disorders |
|  | Glioblastoma, Primary |
|  | Glioblastoma, Secondary |
|  | Astrocytoma |
|  | Vascular Endothelial Cell Vasodilation in Brain |
|  | CCL2 Role in CGRP Expression and Release |
|  | Dopamine Synthesis and Release in Migraine |
|  | Vascular Smooth Muscle Cell Vasodilation in Brain |
|  | Postsynaptic Neuron Activation |
|  | GABRB3 Mutation and Adenosine Deaminase Polymorphism |
|  | Hypocretin Elevation and Monoamine Oxidase Decrease/Inhibition |
|  | Brain Cell Necrosis in Vascular Dementia |
|  | Angiogenesis in Endometriosis |
|  | Coagulation Factors and PAR1/2 Receptors (F2R/F2RL1) in Endometriosis |
|  | EGFR and PTAFR Signaling in Ovarian Cancer |
|  | MIF Role in Cholesteatoma |
|  | Cholesteatoma |
|  | HPV E5 Regulates EDNRA and EGFR Signaling Pathway in Keratinocytes |
|  | Prostate Cancer |
|  | TRPM8 Effects in Prostate Cancer (Hypothesis) |
|  | Ca2+ Toxicity in Lens Cells |
|  | Diabetes Induced Cataract |
|  | VEGFA in Neovascular Glaucoma |
|  | PGF2-alpha Effect on Treatment |
|  | Serotonin Signaling in the Ciliary Body |
|  | Alveolar Macrophages Dysfunction in COPD |
|  | Muscarinic Receptor-Mediated Airway Smooth Muscle Contraction |
|  | VEGFA Low Expression Level in Chronic Bronchitis |
|  | VEGFA High Expression Level in Pulmonary Emphysema |
|  | Alveolar Epithelial Cell Dysfunction in COPD |
|  | Penile Detumescense Augmentation Mechanisms |
|  | Plasmin Role in Glomerulonephritis |
|  | Mesangial Cell Dysfunction in Glomerulonephritis |
|  | Sertoli Cells Dysfunction Causes Male Infertility |
|  | CD38/CD3 -> JUN/FOS/NF-kB Signaling in T-cell Proliferation |
|  | CD38/CD19 -> JUN/FOS/NF-kB Signaling in B-cell Proliferation |
|  | CD38 -> cADPR/Calcium Signaling |
|  | CD157 -> cADPR/Calcium Signaling |
|  | ANGPT1 -> CREB/CREBBP Expression Targets |
|  | ANGPT2 -> AP-1/CREBBP/MYC Expression Targets |
|  | AREG -> NFATC Expression Target |
|  | AREG -> CREB/CREBBP Expression Targets |
|  | AREG -> NCOR2 Expression Targets |
|  | AREG -> SMAD1 Expression Target |
|  | AREG -> HIF1A Expression Targets |
|  | AREG -> AP-1 Expression Targets |
|  | Noradrenaline/Gs Expression Targets |
|  | Noreadrenaline/Gq Expression Targets |
|  | Epinephrine/Gs Expression Targets |
|  | Acetylcholine Expression Targets |
|  | Epinephrine/Gq Expression Targets |
|  | Dopamine/Gi Expression Targets |
|  | B-Cell Receptor -> AP-1 Signaling |
|  | B-Cell Receptor -> NF-kB Signaling |
|  | BTC -> NFATC Expression Targets |
|  | BTC -> AP-1/ATF/CREB Expression Targets |
|  | BTC -> EP300/ETS/ETV/SP1 Expression Targets |
|  | SELE -> ELK-SRF Signaling |
|  | CD19 Expression Targets |
|  | CD81 -> AP-1 Expression Targets |
|  | CD247 Expression Targets |
|  | gamma Globulins Expression Targets |
|  | FCGR3A Expression Targets |
|  | CD22 -> NF-kB Expression Targets |
|  | CD19/CR2 Expression Targets |
|  | CD72 -> CREB/CREBBP Expression Targets |
|  | CD72 -> NF-kB Expression Targets |
|  | CD72 -> STAT Expression Target |
|  | CD8 -> ATF/CREB/CREBBP Expression Targets |
|  | CD8 -> NF-kB Expression Targets |
|  | CD8 -> STAT Expression Targets |
|  | CD80 -> STAT Expression Targets |
|  | CD80 -> NF-kB Expression Targets |
|  | CD80 -> NFATC Expression Targets |
|  | CD86 -> NFATC Expression Targets |
|  | CD86 -> STAT Expression Targets |
|  | CD86 -> NF-kB Expression Targets |
|  | CXCL6 Expression Targets |
|  | CXCL1 Expression Targets |
|  | CXCL2 Expression Targets |
|  | CXCL3 Expression Targets |
|  | CXCL5 Expression Targets |
|  | Inositol Phosphate Metabolism |
|  | Complement Component GPCRs Signaling |
|  | Doxorubicin Induced Cardiotoxicity |
|  | Clozapine Induced Granulocytopenia |
|  | Cyclosporine Induced Nephrotoxicity |
|  | EGF -> HIF1A Expression Targets |
|  | EGF -> AP-1/ATF Expression Targets |
|  | EGF -> CREB/CREBBP/ELK/SRF/MYC Expression Targets |
|  | EGF -> MEF/MYOD/NFATC Expression Targets |
|  | EGF -> TP53 Expression Targets |
|  | EGF -> NCOR2 Expression Target |
|  | EGFR -> NCOR2 Signaling |
|  | EGFR/ERBB3 -> MEF/MYOD/NFATC/MYOG Signaling |
|  | EGFR/ERBB2 -> TP53 Signaling |
|  | ERBB2/3 -> EP300/ETS/ETV/SP1 Signaling |
|  | EGFR -> SMAD1 Signaling |
|  | EGFR/ERBB2 -> HIF1A Signaling |
|  | EGFR -> AP-1/ATF2 Signaling |
|  | EGFR -> AP-1/CREB/ELK/SRF/MYC Signaling |
|  | EREG -> CREB Expression Target |
|  | EREG -> EP300/SP1 Expression Targets |
|  | EREG -> HIF1A Expression Target |
|  | EREG -> AP-1/ATF Expression Targets |
|  | Erythropoietin -> AP-1/MYC/CREB Expression Targets |
|  | Erythropoietin -> ELK/SRF Expression Targets |
|  | F2 -> AP-1/CREB/ELK/SRF/SP1 Expression Targets |
|  | PLG -> AP-1/CREB/ELK/SRF/SP1 Expression Targets |
|  | FGF9 -> AP-1/CREB/MYC Expression Targets |
|  | FGF10 -> AP-1/CREB/CREBBP/MYC Expression Targets |
|  | FGF1 -> AP-1/CREB/ELK/SRF/MYC Expression Targets |
|  | FGF1 -> NCOR2 Expression Target |
|  | FGF7 -> RUNX Expression Targets |
|  | FGF8 -> AP-1/CREB/MYC Expression Targets |
|  | FGF1 -> RUNX Expression Targets |
|  | FGF18 -> AP-1/CREB Expression Targets |
|  | FGF23 -> NCOR2 Expression Targets |
|  | FGF8 -> RUNX Expression Targets |
|  | FGF9 -> RUNX Expression Targets |
|  | FGF2 -> NCOR2 Expression Targets |
|  | FGF4 -> AP-1/MYC Expression Targets |
|  | FGF7 -> AP-1/CREB/CREBBP/MYC Expression Targets |
|  | FGF2 -> RUNX Expression Targets |
|  | FGF2 -> AP-1/CREB/CREBBP/ELK/SRF/MYC Expression Targets |
|  | FGFR -> RUNX2 Signaling |
|  | FGFR -> AP-1/CREB/CREBBP/ELK/SRF/MYC Signaling |
|  | FPR1 -> Cytoskeleton Signaling |
|  | ADCYAP1 Expression Targets |
|  | FSHR Expression Targets |
|  | Glutamate/Gq Expression Targets |
|  | VIP Expression Targets |
|  | HBEGF -> AP-1/ATF Expression Targets |
|  | HBEGF -> TP53 Expression Targets |
|  | HBEGF -> EP300/ETS/ETV/SP1 Expression Targets |
|  | HBEGF -> HIF1A Expression Targets |
|  | HBEGF -> MEF/MYOD Expression Target |
|  | HBEGF -> CREB/MYC Expression Targets |
|  | HGF -> AP-1/CREB/ELK/SRF/MYC Expression Targets |
|  | HGFR -> AP-1/CREB/MYC Signaling |
|  | EGFR Transactivation in Cancer and Non-Cancer Cells |
|  | TAM Receptor Signaling Activation in Cancer |
|  | G0/G1 Cell Cycle Phase Transition Activation in Cancer |
|  | NFKB Canonical Signaling Activation in Cancer |
|  | VEGF Independent Angiogenesis in Cancer |
|  | CDH2 Activation Promotes Cancer Cell Migration and Survival |
|  | Lipogenesis/Lipolysis Activation in Cancer Cells and Cancer-Associated Adipocytes |
|  | Effector T-cell Inactivation in Cancer Immune Escape |
|  | CD19 -> JUN/ELK Signaling |
|  | FcIgER -> ELK-SRF Signaling |
|  | ICAM1 -> AP-1/CREB/ELK/SRF Signaling |
|  | FibronectinR -> AP-1/ELK/SRF/SREBF Signaling |
|  | CTGF -> AP-1/CREB/MYC Expression Targets |
|  | CTGF -> NCOR2 Expression Target |
|  | ICAM1 Expression Targets |
|  | IL16 -> AP-1 Expression Targets |
|  | IL16-> ATF/CREB/CREBBP Expression Target |
|  | IL16 -> NF-kB Expression Targets |
|  | UrokinaseR -> ELK/SRF Signaling |
|  | PTPRC -> BCL6 Expression Targets |
|  | Prostaglandin F Expression Targets |
|  | Thromboxane A2 Expression Targets |
|  | LPA Expression Targets |
|  | PAF/Gq -> AP-1/ATF1/CREB/ERK/SRF Expression Targets |
|  | Dronabinol/Anandamide Expression Targets |
|  | Morphine Expression Targets |
|  | PAF/Gq -> NF-kB Expression Targets |
|  | S1P Expression Targets |
|  | PGE1 Expression Targets |
|  | Metabolism of Triacylglycerols |
|  | Glycosylphosphatidylinositol(GPI)-Anchor Biosynthesis |
|  | Metabolism of Glycerophospholipids and Ether Lipids |
|  | NRG1 -> MEF/MYOD Expression Targets |
|  | NRG1 -> EP300/ETS/ETV/SP1 Expression Targets |
|  | NRG1 -> CREB/CREBBP/ELK/SRF/MYC Expression Targets |
|  | NRG1 -> TP53 Expression Target |
|  | NRG1 -> AP-1/ATF Expression Targets |
|  | NRG1 -> HIF1A Expression Target |
|  | NGF -> SMAD3/NF-kB Expression Targets |
|  | NGF -> AP-1/TP53/MYC Expression Targets |
|  | NGF -> FOXO/MYCN/ELK-SRF Expression Targets |
|  | BDNF Expression Targets |
|  | NTF3 Expression Targets |
|  | NTF4 Expression Targets |
|  | NGF -> CREB/CEBPB/MEF2A Expression Targets |
|  | Adenosine Expression Targets |
|  | PDGFB -> AP-1/CREB/MYC Expression Target |
|  | PDGFC -> CREB Expression Target |
|  | PDGFD -> AP-1 Expression Targets |
|  | PDGF -> AP-1/CREB/CREBBP/MYC Expression Targets |
|  | KIT -> MITF Signaling |
|  | PDGFR -> AP-1/MYC Signaling |
|  | AVP/Gs -> CREB/ELK/SRF/AP-1/EGR Expression Targets |
|  | GNRH1 Expression Targets |
|  | NTS Expression Targets |
|  | GAST Expression Targets in GAST Expression Targets |
|  | AGT -> TP53 Expression Targets |
|  | GNRH2 Expression Targets |
|  | OXT Expression Targets |
|  | POMC Expression Targets |
|  | CCL11 Expression Targets |
|  | EDN1 Expression Targets |
|  | AGT -> CREB Expression Targets |
|  | AVP/Gs -> MEF/MYOD/NFATC/MYOG Expression Targets |
|  | NPY Expression Targets |
|  | AVP/Gq -> STAT Expression Targets |
|  | IFNA1/Gq Expression Targets |
|  | AGT -> ELK/SRF Expression Targets |
|  | TAC1 Expression Targets |
|  | AVP/Gq -> MEF/MYOD/NFATC/MYOG Expression Targets |
|  | EDN3 Expression Targets |
|  | AVP/Gq -> CREB/ELK/SRF/AP-1/EGR Expression Targets |
|  | PDCD1 -> ATF/CREB/CREBBP Expression Targets |
|  | PDCD1 -> NF-kB Expression Targets |
|  | PDCD1 -> AP-1 Expression Targets |
|  | PTPRC -> BCL6 Signaling |
|  | NPR1/NPR2 -> Fatty Acid Signaling |
|  | AngiopoietinR -> AP-1 Signaling |
|  | GDNF -> HSF1 Signaling |
|  | NTRK -> AP-1/CREB/ELK/SRF/MYC/SMAD3/TP53 Signaling |
|  | GAS6 -> AP-1/CREB Expression Targets |
|  | FIGF -> AP-1 Expression Target |
|  | FIGF -> NCOR2 Expression Target |
|  | KITLG -> AP-1/CREB/CREBBP/MYC Expression Targets |
|  | GDNF -> HSF1 Expression Targets |
|  | CSF1 -> AP-1/CREB/CREBBP/MYC Expression Targets |
|  | PGF -> AP-1/CREB/CREBBP/MYC Expression Targets |
|  | FLT3LG -> AP-1/CREB/CREBBP Expression Targets |
|  | WNT/Calcium non-Canonical Signaling |
|  | Lysophosphatidic Acid/LPARs Signaling |
|  | Ras-GRF Regulation Signaling |
|  | MERTK Signaling |
|  | Ras Signaling |
|  | AXL Receptor Tyrosine Kinase Signaling |
|  | VEGF Signaling |
|  | Estrogens/ESR1 non-Genomic Signaling |
|  | EGFR Signaling |
|  | NF-kB Canonical Signaling |
|  | ERK/MAPK Canonical Signaling |
|  | TCR -> CREB/CREBBP/ATF Expression Targets |
|  | TCR -> NF-kB Expression Targets |
|  | TCR -> NFAT Expression Targets |
|  | TCR -> AP-1 Expression Targets |
|  | T-Cell Receptor -> AP-1 Signaling |
|  | T-Cell Receptor -> NF-kB Signaling |
|  | T-Cell Receptor -> NFATC Signaling |
|  | T-Cell Receptor -> CREBBP Signaling |
|  | TGFA -> TP53 Expression Targets |
|  | TGFA -> HIF1A Expression Targets |
|  | TGFA -> MEF/MYOD/NFATC Expression Targets |
|  | TGFA/AP-1/ATF Expression Targets |
|  | TGFA -> CREB/CREBBP/ELK-SRF/MYC Expression Targets |
|  | Thrombopoietin -> AP-1/CREB/CREBBP/MYC Expression Targets |
|  | IL1B -> PGE2 Expression Targets |
|  | ROS in Neutrophil Mediated Cell Damage |
|  | ROS in Triggering Vascular Inflammation |
|  | ROS and RNS in Vasoconstriction and Vasodilation Regulation |
|  | Ca2+ Cytosolic Overload |
|  | ROS in Angiotensin Mediated Cardiovascular Remodeling and Hypertrophy |
|  | PLAU -> ELK-SRF/AP-1 Expression Targets |
|  | VEGFA -> ATF/CREB/ELK-SRF Expression Targets |
|  | VEGFA -> NFATC Expression Targets |
|  | VEGFC -> ATF Expression Target |
|  | VEGFA -> AP-1/CREBBP/MYC Expression Targets |
|  | VEGFA -> NCOR2 Expression Target |
|  | VEGFR -> NFATC Signaling |
|  | VEGFR -> AP-1/CREB/MYC Signaling |
|  | VEGFR -> ATF/CREB/ELK-SRF Signaling |
|  | ace2 |
|  | Model of PPARG signaling in psoriasis |
|  | New Pathway |
|  | 2\_Hyperseborrhea in acne vulgaris |
|  | 2\_Acute phase initiation in atopic dermatitis |
|  | 1\_The onset of atopic dermatitis |
|  | 2\_Podocyte dysfunction in glomerulonephritis |
|  | 3\_Enhanced angiogenesis in endometriosis |
|  | 1\_Disruption of luteinizing hormone and follicle-stimulating hormone secretion in PCOS |
|  | 2\_Diabetes-induced cataract |
|  | 2\_Pathogens stimulate mucins expression in the middle ear |
|  | 2\_Cardiomyocyte hypertrophy |
|  | 3\_Dysfunction of smooth muscle cells in hypertension |
|  | 4\_Endothelial cells repress vasoconstriction in hypertension |
|  | 1\_Rise in aldosterone synthesis in adrenal zona glomerulosa cells |
|  | 2\_The intense proliferation and contractile activity of smooth muscle cells in pulmonary hypertension |
|  | 1\_Cigarette smoke causes dysfunction and production of inflammatory mediators in alveolar epithelial cells |
|  | 3\_1\_Mucus accumulation in asthma |
|  | 2\_1\_Eosinophilia and neutrophilia in asthma: eosinophil migration and activation |
|  | 2\_2\_Eosinophilia and neutrophilia in asthma: eosinophil apoptosis |
|  | 4\_1\_Airway smooth muscle cells contraction |
|  | 4\_2\_Airway smooth muscle cells proliferation and airway tissue remodeling |
|  | 4\_Mucin hyperproduction in goblet and mucous cells in COPD |
|  | 1\_Triglyceride storage in NAFLD |
|  | 2\_CD8+ T-cell response and mucosal damage in celiac disease |
|  | 1\_2\_Insulin synthesis and secretion: insulin secretion |
|  | 1\_1\_Insulin synthesis and secretion: insulin processing and storage |
|  | 4\_3\_Insulin resistance: FFA-related insulin resistance |
|  | 1\_CFTR expression and degradation in epithelial cells in cystic fibrosis (class I-II mutations) |
|  | 1\_Microangiopathy in diabetic neuropathy |
|  | 3\_Pain perception in diabetic neuropathy |
|  | 2\_Neuron and Schwann cell death |
|  | 1\_1\_Glutamate overload enforced prostaglandins and NO synthesis in neurons |
|  | 1\_2\_Glutamate overload and NO-mediated vasodilation |
|  | 1\_Decreased secretion of thyroid hormones |
|  | 2\_1\_General pathways of thyroid hormone signaling |

---

|  |  |
| --- | --- |
| Source | Biopax |
|  | AraCyc |

---

|  |  |
| --- | --- |
| MedScan ID | 1808946 |

---

|  |  |
| --- | --- |
| Alias | [3H]1,2-diacylglycerol |
|  | 1,2-glyceryl diesters |
|  | D-1,2-Diacylglycerol |
|  | glycerol diacylate |
|  | [11C]diacylglycerol |
|  | [18F]diacylglycerol |
|  | 1,2-sn-diacylglycerols |
|  | DAG |
|  | 1,2-Diacylglycerol |
|  | 1,2-glyceryl diester |
|  | sn-1,2-diacylglycerol |
|  | 1,2-diacyl-sn-glycerols |
|  | diacylglyceride |
|  | Diglycerides |
|  | [14C]diacylglycerol |
|  | 1,2-sn-diacylglycerol |
|  | Diglyceride |
|  | diacylglycerols |
|  | 2,3 diacyl sn glycerol |
|  | diacylglycerol |
|  | [3H]diacylglycerol |
|  | 1,2-diacyl-sn-glycerol |
|  | 1,2 diglyceride |
|  | 1,2-Diacetylglycerol |
|  | a D-1,2-diacylglycerol |
|  | 1,2-diasylglycerol |
|  | a 1,2-diacyl-sn-glycerol |

---

|  |  |
| --- | --- |
| Reaxys ID | 18700847 |
|  | 14509969 |
|  | 7096022 |

---

|  |  |
| --- | --- |
| PubChem SID | 2633 |

---

|  |  |
| --- | --- |
| Patent ID | WO2008/148549 |
|  | US2006/30537 |

---
